# Human amniotic fluid-derived and dental pulp-derived stem cells seeded into collagen scaffold repair critical-size bone defects promoting vascularization

**DOI:** 10.1186/scrt203

**Published:** 2013-05-21

**Authors:** Tullia Maraldi, Massimo Riccio, Alessandra Pisciotta, Manuela Zavatti, Gianluca Carnevale, Francesca Beretti, Giovanni B La Sala, Antonella Motta, Anto De Pol

**Affiliations:** 1 Department of Surgical, Medical, Dental and Morphological Sciences with interest in Transplant, Oncology and Regenerative Medicine, University of Modena and Reggio Emilia, Via Del Pozzo 71, 41100 Modena, Italy; 2 Department of Obstetrics and Gynecolgy, Arcispedale Santa Maria Nuova, V. le Risorgimento 80, Reggio Emilia 42100, Italy; 3 BIOtech Research Center and European Institute of Excellence on Tissue Engineering and Regenerative Medicine, University of Trento, Via Mesiano 77, 38123 Trento, Italy

**Keywords:** Bone, Collagen scaffold, Regenerative medicine, Stem cells, Vascularization

## Abstract

**Introduction:**

The main aim of this study is to evaluate potential human stem cells, such as dental pulp stem cells and amniotic fluid stem cells, combined with collagen scaffold to reconstruct critical-size cranial bone defects in an animal model.

**Methods:**

We performed two symmetric full-thickness cranial defects on each parietal region of rats and we replenished them with collagen scaffolds with or without stem cells already seeded into and addressed towards osteogenic lineage *in vitro*. After 4 and 8 weeks, cranial tissue samples were taken for histological and immunofluorescence analysis.

**Results:**

We observed a new bone formation in all of the samples but the most relevant differences in defect correction were shown by stem cell–collagen samples 4 weeks after implant, suggesting a faster regeneration ability of the combined constructs. The presence of human cells in the newly formed bone was confirmed by confocal analysis with an antibody directed to a human mitochondrial protein. Furthermore, human cells were found to be an essential part of new vessel formation in the scaffold.

**Conclusion:**

These data confirmed the strong potential of bioengineered constructs of stem cell–collagen scaffold for correcting large cranial defects in an animal model and highlighting the role of stem cells in neovascularization during skeletal defect reconstruction.

## Introduction

Over 2 million bone replacement procedures are performed every year worldwide requiring the use of bone graft materials. This makes bone second only to blood on the list of transplanted materials. With demand escalating and increasing limitations with traditional bone graft supply, techniques are being developed to provide alternatives with properties suitable for clinical use [1-4].

A critical-size bone defect will not heal spontaneously and tissue engineering strategies are a very promising option [5]. For this strategy, therapeutic scaffolds have been developed from various classes of natural materials including collagen, fibroin [6] and hyaluronic acid-based hydrogel [7]. Indeed, cell-free scaffolds as tissue graft substitutes have been used in the human clinical setting since Yannas and colleagues developed the collagen glycosaminoglycan scaffold for skin grafting [8,9].

Among the natural-derived polymers, collagen – the most abundant protein in the body and the major component of the extracellular matrix – is regarded as one of the most useful biomaterials for growth factor delivery owing to its excellent biocompatibility and safety [10]. On the other hand, some authors reported that collagen scaffolds undergo deformation leading to an implant compression within the defect or are even displaced beyond the borders of the defect site [11]. However, most of materials possessing the needed mechanical forces are not optimal for cell growth and differentiation [5].

The potential of cell-free scaffolds to be used as an off-the-shelf product presents an ideal clinical solution to the limitations of traditional bone graft; however, cell-free scaffolds are inadequate for large bone defects and the principal strategies of bone tissue engineering suggest the use of cells seeded onto scaffolds prior to implantation.

Recent investigations have focused on the use of stem cells instead of mature differentiated cell types due to their expansion potential and ease of access. In particular, mesenchymal stem cells have shown promising results in a number of tissue engineering areas; for example, tendon [12,13], cartilage [14], fat [15] and bone [15-17]. Previously, we have demonstrated the osteogenic potential of human dental pulp stem cells (DPSCs) and amniotic fluid stem cells (AFSCs) *in vitro* and *in vivo* in an ectopic model [18,19]. Human DPSCs and AFSCs both present the advantage of easy recruitment without ethical problems; in fact, they can be easily isolated and expanded in culture showing *in vitro* and *in vivo* multipotential or pluripotential plasticity.

Some of the fundamental features of biomaterials thought to be important for effective bone regeneration include: a biochemical composition and structure that supports osteogenic cell responses; appropriate kinetics of biodegradability, without any release of toxic byproducts; and a highly interconnected porous network that allows for proper tissue in growth and vascularization of the biomaterial [20]. Vascularization of the biomaterial is therefore an essential step in tissue healing, as this process provides the nutrients and oxygen needed for bone cells to survive, while facilitating removal of waste products from cell metabolism, avoiding the onset of a necrosis process [21-24].

We have already investigated the stem cells/scaffold construct potential in osteo regeneration *in vivo*, focusing on the analysis of new scaffold applications [18,25]. Here we present an evaluation of the vascularization capability role of human stem cells, derived from alternative sources, during bone reconstruction in animal bone defects. For this purpose we decided to employ a collagen-based scaffold since it is already used in clinical practice and it is suitable in bone regeneration for properties of osteoinductivity and biocompatibility. We chose a cranial defect in order to exclude the lack of stability problem of collagen scaffold.

The specific aims of this study were therefore: to compare the ability of collagen and tissue-engineered constructs seeded with human stem cells, to heal critical-size bone defects using an established rat cranial defect *in vivo* model; and to determine the relationship between the neovascularization and the overall defect healing.

Healing rates were assessed by measuring new bone formation within the defect using histomorphometric techniques. Immunohistochemistry was carried out to analyze the differentiation potential of the tissue-engineered constructs and the cell-free scaffolds.

## Materials and methods

### Cell culture and seeding

Supernumerary amniocentesis samples were provided by the Laboratorio di Genetica, Ospedale Santa Maria Nuova (Reggio Emilia, Italy). All samples were collected with informed consent of the patients according to Italian law and ethical committee guidelines.

AFSCs were isolated as previously described by De Coppi and colleagues [26]. Human amniocentesis cultures were harvested by trypsinization, and subjected to c-Kit immunoselection by MACS Technology (Miltenyi Biotec, Cologne, Germay). Human AFSCs were subcultured routinely at 1:6 dilution and were not allowed to expand beyond the 70% of confluence.

Human DPSCs were isolated as previously described by Riccio and colleagues [27]. Briefly, DPSCs were obtained by magnetic cell sorting using MACS Technology by three successive sortings performed using specific antibodies against: CD34, a marker of stromal and hemopoietic pluripotent stem cells; c-Kit, the tirosin-kinase receptor of stem cells factor; and STRO-1, a stromal stem cell surface marker.

AFSCs and DPSCs were grown in culture medium of αMEM supplemented with 10% fetal bovine serum, 2 mM l-glutamine, 100 U/ml penicillin and 100 μg/ml streptomycin (all Invitrogen, Carlsbad, CA, USA).

Discs of height 1.5 mm and diameter 13 mm were cut from collagen scaffolds (sponges of horse-derived collagen – Condress, Istituto Gentilini, Pisa, Italy) and placed in 24-well tissue culture plates. All scaffolds were then washed twice with culture medium (1 hour for each rinse). Cells, at the third passage, were seeded on each scaffold at density of 1,500 cells/mm^3^ and cultured for 24 hours with 2 ml culture medium. After 24 hours the culture medium was changed for osteogenic medium (culture medium supplemented with 100 nM dexamethasone, 10 mM β-glycerophosphate, 50 μg/ml ascorbic acid-2-phosphate; Sigma-Aldrich, St Louis, MO, USA). Cell/scaffold cultures were then maintained in osteogenic medium for 1 week in an incubator at 37°C with 5% CO_2_ (medium changed twice). Some samples were stained with 6-carboxyfluorescein diacetate (Sigma-Aldrich) to test the viability of seeded cells.

### Surgery and transplantation procedure

For implantation, CD® IG5 male rats with age ranging between 12 and 14 weeks (Charles River Laboratories, Lecco, Italy) were used in the study.

To evaluate the potential of the DPSCs and AFSCs to reconstruct large cranial defects, we performed two symmetric full-thickness cranial defects of 5 mm × 8 mm on each parietal region of 30 animals (see Additional file 1) [18]. The animals were anesthetized with an intraperitoneal injection (0.2 ml/100 g body weight) of ketamine hydrochloride (5%). A midline skin incision was performed from the nose-frontal area to the external occipital protuberance.

The skin and underlying tissues, including the periosteum, were reflected laterally to expose the full extent of the calvaria. The cranial defect was performed with a drill with a micromotor under constant irrigation with sterile physiological solution to prevent overheating of the bone. The underlying dura mater was undisturbed.

One scaffold 5 mm × 8 mm × 1.5 mm size was implanted into each cranial defect and adapted to fill the entire defect area. Each animal received two equal constructs. After the scaffold implantation, the incisions were sutured with prolene 4–0 sutures (Ethicon, Roma, Italy). Animals were immunocompromised using cyclosporine A at a dosage of 15 mg/kg body weight, administered 4 hours before transplantation and then daily for 2 weeks. During the last weeks the daily dosage was reduced gradually up to 6 mg/kg body weight.

The rats were sacrificed 4 and 8 weeks later and the calvariae were rapidly explanted and fixed in 4% paraformaldehyde in PBS for 3 hours. All animal procedures were performed according to the guidelines approved on 19 October 2010 by the Committee of Use and Care of Laboratory Animals of the University of Modena e Reggio Emilia.

All experiments were carried out according to the Bioethical Committee of the Italian National Institute of Health. Animal care, maintenance and surgery were conducted in accordance with Italian Law (D.L. No. 116/1992) and European legislation (ECC No. 86/609).

### Radiography

Explanted calvariae samples were radiographed by a Kodak RVG Digital Radiography System (FORB, Torino, Italy).

### Histology

Histological sections were stained with Alizarin Red staining for light microscopic observation. Fixed samples were decalcified in 0.5 M ethylenediamine tetraacetic acid, pH 8.3. Parietal bones were then rinsed in PBS, dehydrated with graded ethanol, diaphanized and embedded in paraffin. Transversal serial sections (10 μm thick) were cut through the midline of the implant area. Routine H & E staining was performed to analyze morphological details.

Immunohistochemistry was performed using mouse anti-human mitochondrial protein (Millipore, Billerica, MA, USA). Endogenous peroxidase activity was quenched by first incubating sections in 10% methanol and 3% H_2_O_2_, followed by three washes in Tris-buffered saline (pH 7.4), and incubation for 1 hour in 3% BSA in Tris-buffered saline with 0.2% Triton X-100. Sections were incubated overnight in the primary antibody in Tris-buffered saline with 0.2% Triton X-100. Following three 10-minute washes, tissue sections were incubated for 1 hour in peroxidase-labeled anti-mouse (Amersham GE Healthcare, Freiburg, Germany). Following another three washes, staining was visualized using diaminobenzidine (2 mg/ml) and H_2_O_2_ (0.3 μl/ml). Sections were then stained with Harris hematoxylin. Images of histological samples were obtained by a Zeiss Axiophot microscope equipped with polarizer filters and with a Nikon DS-5Mc CCD color camera (Nikon Instruments, Japan).

### Confocal microscopy

Histological sections were processed as previously described by Riccio and colleagues [27]. Mouse anti-human mitochondrial protein (diluted 1:100; Millipore), rabbit anti-von Willebrand (diluted 1:50; Sigma Aldrich), and osteocalcin (diluted 1:50; Millipore) in PBS containing 3% BSA for 1 hour at room temperature were used as primary antibodies. Secondary antibody was diluted 1:200 in PBS containing 3% BSA (goat anti-mouse Alexa 647, goat anti-rabbit Alexa 546). After washing in PBS, samples were stained with 1 μg/ml 4′,6-diamidino-2-phenylindole in H_2_O for 1 minute and then mounted with anti-fading medium (0.21 M 1,4-diazabicyclo[2.2.2]octane and 90% glycerol in 0.02 M Tris, pH 8.0). Negative controls consisted of samples not incubated with the primary antibody.

Confocal imaging was performed on a Nikon A1 (Nikon Instruments, Japan) confocal laser scanning microscope as described previously [28]. Spectral analysis was carried out to exclude overlapping between two signals or the influence of autofluorescence background on the fluorochrome signals. The confocal serial sections were processed with ImageJ software to obtain three-dimensional projections, as described previously [18]. The image rendering was performed by Adobe Photoshop software (Adobe Systems Software, Ireland).

### Statistical analysis

*In vitro* experiments were performed in triplicate. For *in vivo* experiments each treatment was performed twice (two cranial defects) in five animals. For quantitative comparisons, values were reported as mean ± standard deviation based on triplicate analysis for each sample. To test the significance of observed differences between the study groups, analysis of variance with *post-hoc* Bonferroni correction was applied. *P* <0.05 was considered statistically significant.

## Results

**Figure 1:**
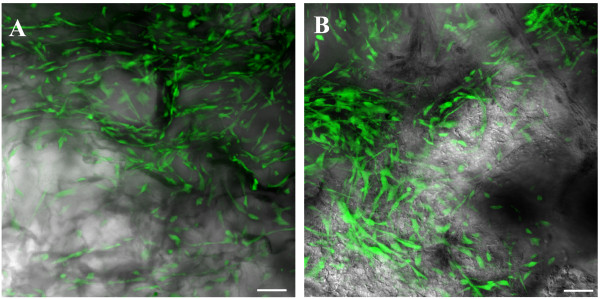
**Microscopic analysis of dental pulp and amniotic fluid stem cells cultured in collagen scaffolds.** 6-Carboxyfluorescein diacetate-stained cells present in (**A**) dental pulp stem cell–collagen and (**B**) amniotic fluid stem cell–collagen constructs after 7 days of culture were analyzed by *in vivo* confocal microscopy. Confocal images of cells (green) were superimposed to differential interference contrast images of scaffolds (gray). Scale bar = 20 μM.

To study the bone-forming ability of AFSCs and DPSCs on collagen scaffolds, a critical-size bone defect was obtained by a full-thickness dissection removing both the internal and external tables of compact bone and the trabecular diploë constituting the parietal skeletal segment. The stem cell–scaffold constructs were induced in osteogenic medium for 7 days, in order to have a cell population committed to osteogenic differentiation yet able to proliferate. After 1 week of osteogenic medium exposure, expression of the typical markers for osteodifferentiation, such as osteocalcin, osteopontin and osterix, is not different from nonexposed AFSCs and DPSCs [19,27]. Tests performed with the 6-carboxyfluorescein diacetate probe demonstrated that, 7 days after the cell seeding into the scaffold before implant, scaffolds are colonized by viable cells. In order to check the cell presence inside the scaffold, confocal analysis of 100 μm collagen sponge thickness was performed and is shown in Figure 1. Further, both cell types are present and homogeneously distributed within the micro-cavities delineated by the scaffold architecture and no difference in the cell density was observed.

Constructs were than implanted into a critical-size parietal defect for up to 8 weeks. None of the animals died of infection or any other complication.

### Radiological analysis

**Figure 2:**
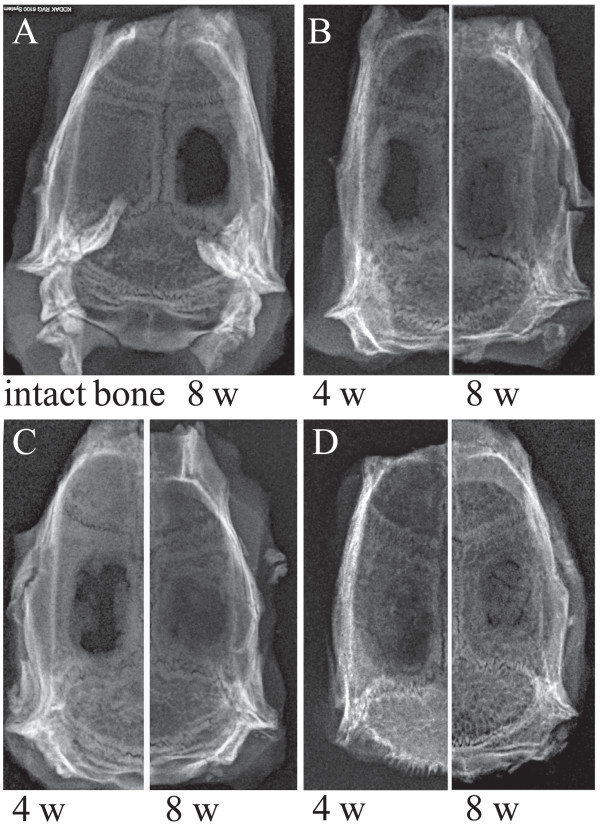
**Radiological analysis of rat calvariae dissected 4 or 8 weeks after surgery.** Images are representative of (**A**) intact bone (left panel) and the bone healing in the cranial defects (right panel), (**B**) scaffolds alone, and scaffold seeded with (**C**) dental pulp stem cells or (**D**) amniotic fluid stem cells.

Radiography scans of *ex vivo* bone explants demonstrate qualitatively and quantitatively mineralized tissue levels in the defect (Figure 2).

Most notably, the empty control group remains largely void of mineralized tissue (Figure 2A, right) and the unseeded collagen group shows progressive infilling with mineralized tissue (Figure 2B) showing low to moderate rates of bone growth in the defect. After 4 weeks, AFSC-seeded samples show the best bone formation compared with the other groups. Both AFSC and DPSC seed scaffold groups (Figure 2C,D) showed almost complete bridging of the defect by 8 weeks. This suggests a higher mineralized tissue volume in the defect treated with the cell-seeded scaffolds compared with the cell-free collagen sponge.

### Qualitative histology

To evaluate the parietal bone reconstructing process by histological analysis, serial sections of the middle line of the parietal bone defect containing the implants were stained with H & E. Samples were then observed by white field and polarized light microscopy.

**Figure 3:**
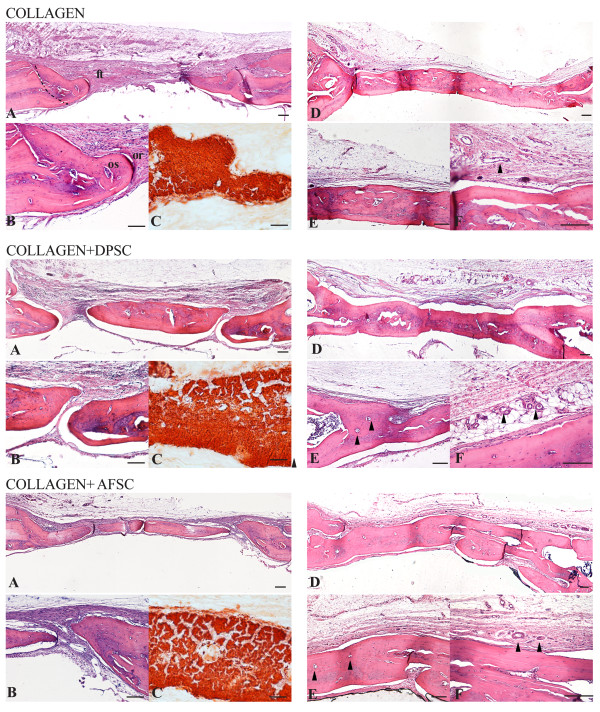
**Comparative histologic analysis of cranial defect reconstruction 4 and 8 weeks post surgery.** Comparative histologic analysis of cranial defect reconstruction by collagen, collagen + dental pulp stem cell (DPSC) or collagen + amniotic fluid stem cell (AFSC) constructs 4 and 8 weeks post surgery. (**A**) H & E staining of cranial defects closed with constructs 4 weeks after implant (magnified in (**B**) and (**C**) staining for Alizarin red. (**D**) Representative images showing samples 8 weeks after implant (magnified in (**E**), (**F**). Dotted line defines (left) the host bone and (right) the new bone. Arrowheads indicate vessels. Scale bar = 100 μM. ft, fibrous tissue; or, osteoblasts; os, osteoid.

Figure 3 presents representative photomicrographs of engineered osseous grafts *in vivo*. The presence of vessels in the scaffold area indicates that all implanted constructs have been successfully vascularized in unmineralized tissue (as indicated by arrows in Figure 3, collagen, collagen+DPSC, collagen+AFSC F) and in mineralized tissues in (as indicated by arrows in Figure 3, collagen+DPSC, collagen+AFSC E).

The bone defects not treated with implant, after 4 weeks, appear filled with structurally and morphologically organized fibrous tissue consistent with failure to heal the bone defect (shown in [18]), demonstrating the critical size of the defect. After 4 weeks, in the collagen scaffold alone implant, fibrous tissue extends from the defect margin (Figure 3, collagen A) with a few small areas of new bone formation visible immediately adjacent to the host bone (to the left of the dotted line in Figure 3A), indicating unsuccessful efforts at regeneration. However, the sample presented mineralized matrix and calcium deposition, as noted by Alizarin staining (Figure 3, collagen C). Some areas of unfilled defect were still occupied by remnant of the scaffold implanted at surgery (Figure 3, collagen A,B). A rim of osteoblasts exhibited a dense arrangement at the inner edge of the newly formed woven bone (Figure 3 collagen B), with osteoid forming in advance of this. After 8 weeks of implant, collagen scaffold filled defects did exhibit much more complete signs of healing (as demonstrated in Figure 3, collagen D,E,F; see Additional file 2). However, the bone regeneration occurs prevalently in the scaffold layers directly in contact with the outer surface of the dura mater.

The presence of human stem cells dramatically improves the restoration power of collagen scaffold (Figure 3, collagen+DPSC, collagen+AFSC), as shown before by radiograph analysis. With both stem cell types, we can observe that the defect is partially recovered by newly formed bone tissue that appears discontinuous and thinner than pre-existing bone after 4 weeks. In fact, the margins of newly formed bone were unconnected with the pre-existing bone while, between them, connective tissue was present (Figure 3, collagen+DPSC A, collagen+AFSC A). In particular, the AFSC sample showed a better recovering performance (Figure 3, collagen+AFSC A) compared with the DPSC sample (Figure 3, collagen+DPSC A). Moreover, Alizarin red analysis confirmed the characteristics of bone regeneration inside the defects (Figure 3, collagen+DPSC C, collagen+AFSC C). After 8 weeks, however, the bone defects appear almost completely filled in both bone layers (Figure 3, collagen+DPSC D, collagen+AFSC D; see Additional file 2).

The presence of a vast network of vessels is clearly visible in the not yet mineralized construct area (Figure 3, collagen F, collagen+DPSC F, collagen+AFSC F).

### Quantitative histomorphometry

**Figure 4:**
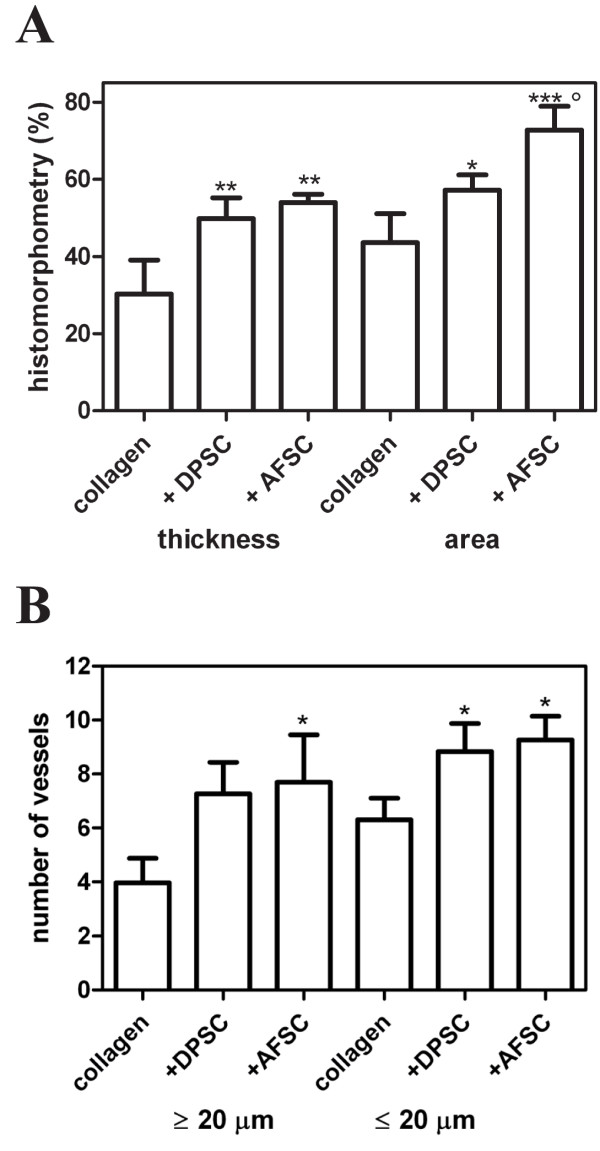
**Quantitative histomorphometry for histologic analysis of cranial defect reconstruction by collagen constructs 4 weeks post-surgery.** (**A**) Percentage of reconstructed normal thickness and area of parietal bone in the three different groups. (**B**) Average number of vessels present in 0.12 mm of implant. Values, calculated on three slices of each of the 10 specimens for group, are expressed as mean ± standard deviation. *P ≤0.05, **P ≤0.01, ***P ≤0.001 versus collagen group; °*P* ≤0.05 versus collagen+DPSC group. AFSC, amniotic fluid stem cell; DPSC, dental pulp stem cell.

Quantitative histomorphometry was carried out to determine the levels of healing in the different groups after 4 weeks. Quantitative values of thickness (Figure 4A) were obtained by measuring at 10 different points the thickness of the new bone for each sample and normalizing the average obtained to the respective thickness of squamosal suture (suture connecting the temporal bones to the parietal bones) (*TSO*). The average of all 10 samples is shown in Figure 4A. We also compared the ratio obtained between the regenerated bone area value (*AR*) and the pre-existing parietal area value (*AO*). The normal area (*AO*) of each sample was calculated considering the area value of the nonoperated sample (*AC*) and the thickness of its squamosal suture (*TSC*). For each samples, the pre-existing area value (before the operation, *AO*) is obtained through the following ratio: *TSC*:*TSO* = *AC*:*AO.*

The cell-free group showed mineralized tissue levels in the defect of around 30%, and the cell-seeded groups were significantly higher. In particular, we also observed a significant difference between AFSC and DPSC area reconstruction, suggesting that the AFSC/collagen construct has the highest potential.

**Figure 5:**
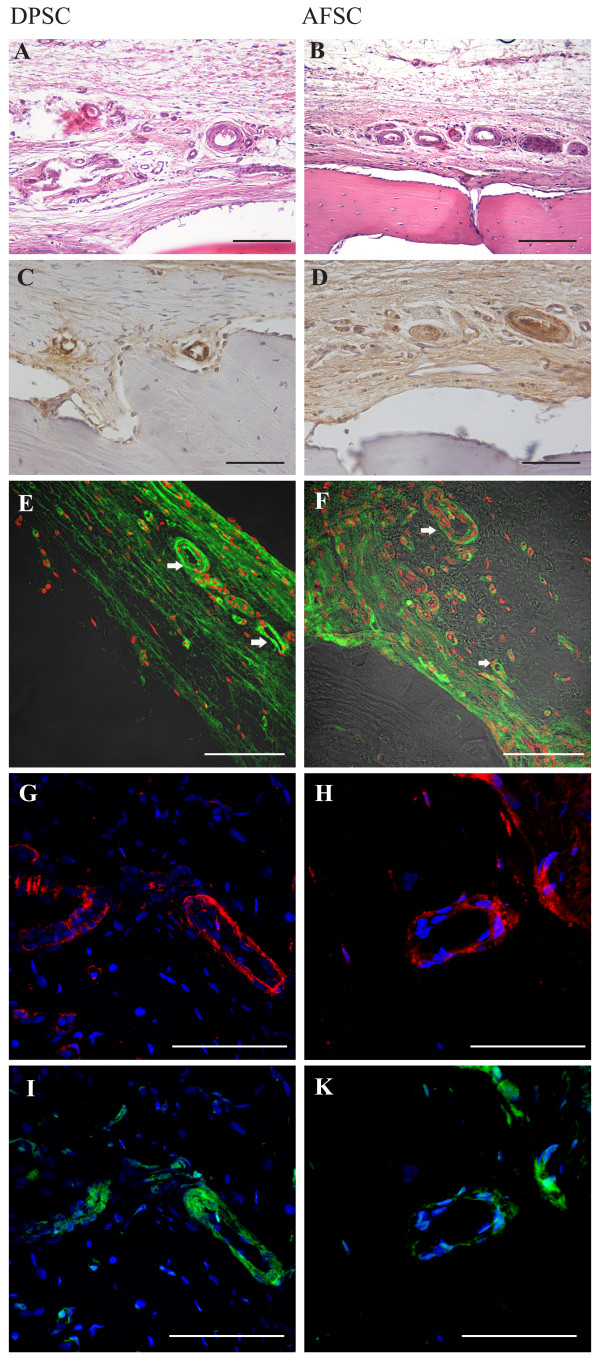
**Presence of human stem cells in vessels 8 weeks after surgery.** H & E staining of cranial defects (**A**) closed with collagen + dental pulp stem cells (DPSC) and (**B**) closed with collagen + amniotic fluid stem cells (AFSC). Scale bar = 100 μM. Diaminobenzidine staining of cranial defects (**C**) closed with collagen + DPSC and (**D**) closed with collagen + AFSC. Scale bar = 100 μM. Confocal images of implants obtained 8 weeks after surgery. Double fluorescence signals from 4’,6-diamidino-2-phenylindole (DAPI; red) and anti-human mitochondria protein (green) antibody images: (**E**) collagen colonized with DPSC, (**F**) collagen colonized with AFSC. Triple fluorescence signals from DAPI (blue) and anti-human mitochondria protein (red) antibody images or DAPI (blue) and anti-von Willebrand (green) antibody images: (**G**), (**I**) collagen colonized with DPSC, (**H**), (**K**) collagen colonized with AFSC. Scale bar = 50 μM.

In parallel we carried out quantitative counting of vessels present in the collagen scaffold not yet modified in mineralized tissue, in order to compare the three different groups also for their new vascularization potential. In fact, the number of vessels inside the new bone does not differ among the three types of constructs (data not shown). However, as shown in Figure 4B, cell-seeded groups clearly present a higher number of vessels, both capillary and bigger vessels (a capillary being considered a vessel with diameter <20 μm). The collagen group demonstrated tiny areas of new vessel formation at the defect margin, reaching 4 ± 2.5 in the defect area. The cell-seeded groups demonstrated an ability to stimulate vascularization, since the ratio between the samples with cells and those without is >1.5 for both cell types and the vessel dimension considered. In fact, Figure 5A,B clearly shows the presence of several arterioles with diameter around 50 μm in samples seeded with DPSCs and AFSCs. This suggests a possible connection between a higher amount of vessels and mineralized tissue in the defect treated with the cell scaffolds compared with the empty defect and the collagen groups.

### Immunohistochemistry

**Figure 6:**
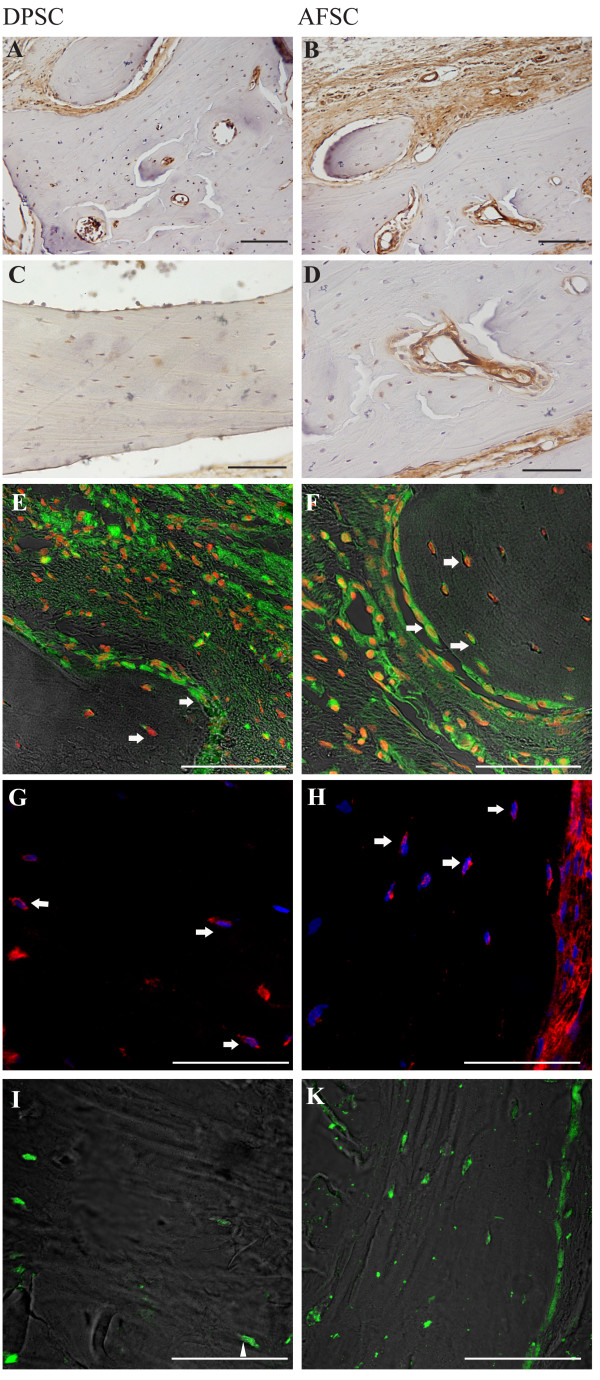
**Presence of human stem cells in the new bone 8 weeks after surgery.** Diaminobenzidine staining of cranial defects (**A**), (**C**) closed with collagen + dental pulp stem cells (DPSC) and (**B**), (**D**) closed with collagen + amniotic fluid stem cells (AFSC). Scale bar = 100 μM. Confocal images of implants obtained 8 weeks after surgery. Double fluorescence signals from 4’,6-diamidino-2-phenylindole (DAPI; red) and anti-human mitochondria protein (green) antibody images: (**E**) collagen colonized with DPSC, (**F**) collagen colonized with AFSC. Triple fluorescence signals from DAPI (blue) and anti-human mitochondria protein (red) antibody images or DAPI (blue) and anti-osteocalcin (green) antibody images: (**G**), (**I**) collagen colonized with DPSC, (**H**), (**K**) collagen colonized with AFSC. Scale bar = 50 μM.

The aim of this part of the study was to establish the cell nature of the osteo and vascular tissue in engineered constructs. Based on our recent work [18], cells inside the new bone were still of human origin and not from the host, after 4 weeks of implants. We demonstrate this issue by four different approaches: RT-PCR analysis (see Additional file 3), fluorescence hybridation *in situ* analysis (see Additional file 4), immunofluorescence experiments investigating the presence of human nuclei (see Additional file 5), and human mitochondria (reported below). This was possible by staining the *ex vivo* specimens for with anti-human mitochondria, that recognizes only mitochondrial protein from human origin. Both immunohistochemistry (Figures 5C,D and 6A,B,C,D) and immunofluorescence analyzed with a confocal microscope (Figures 5E,F and 6E,F) demonstrate the presence of human cells in cell–collagen implants.

In control samples (collagen alone; data shown in Additional file 6) only 4′,6-diamidino-2-phenylindole staining occurs, while human mitochondria are not detectable. In Figure 6E,F the images of stem cell–scaffold implants demonstrate that most cells are clearly labeled by anti-human mitochondria antibody, indicating survival of the donor cells *in vivo* also after 8 weeks of implant. In particular, Figure 6E,F shows that human cells are present around and inside lamellar tissue in growth, as indicated by arrows, representing the osteoblast mineralization front and osteocytes included in the mineralized matrix.

Double staining with anti-human mitochondria and anti-osteocalcin, produced by osteoblasts and therefore used as a marker for the bone formation process, was then performed. Figure 6G,H,I,J,K shows that cells of human origin (stained in red in Figure 6G,H) are also positive for osteocalcin (green signal in Figure 6I,K), confirming the functionality of the osteo-differentiated human stem cells *in vivo*.

The same approach demonstrates that all new vessels, some of them indicated by arrows in the figures, are completely surrounded by an endothelium composed of cells from human origin (Figure 5C,D,E,F). In fact, cells labeling anti-von Willebrand (Figure 5G,H), a protein produced constitutively in the endothelium, are also positive for human mitochondria (Figure 5I,K).

In fact the capability of DPSCs and AFSCs to differentiate in vascular tissue *in vitro* both autonomously and after vascular endothelial growth factor exposure has already been demonstrated by several authors [29,30].

## Discussion

Bone is a dynamic, highly vascularized tissue with the unique capacity to heal and to remodel without leaving a scar [31]. Large bone defects, caused by trauma, tumor resection, or infections, require implants to restore the loss of function. Current treatments of large bone defects are based on autogene or allogene bone grafts and metal or ceramic implants that still have several limitations. Bone tissue engineering could provide an alternative to conventional treatments of fracture healing. However, this approach requires a matrix to allow progenitor cell delivery and support tissue invasion.

Bone defect repair is one of the major targets of tissue engineering research [32]. Pre-seeding a biomaterial with tissue-specific cells prior to implant is one method that is being examined to speed up tissue regeneration [33]. However, one of the major problems after the implantation of large biomaterials is the slow vascularization of the material [34].

Vascularization is a key process in tissue engineering and regeneration and represents one of the most important issues in the field of regenerative medicine. Thus, several strategies to improve vascularization are currently under clinical evaluation.

In fact, approximately 10% of fractures are slow or nonhealing [35] and may be characterized by poor vascularization and a resulting hypoxic local microenvironment [36]. The absence of a sufficient vasculature impairs native healing processes by limiting the availability of nutrients to resident progenitor cells and infusion of inductive factors from the systemic circulation that promote healing. The consequences of a low-oxygen environment on cells are unclear but may include enhanced apoptosis, reduced proliferation, and inhibited differentiation; all of which ultimately impact tissue repair.

Cell transplantation is a promising alternative to the gold standard of autologous bone grafting to stimulate bone repair. Human DPSCs or AFSCs are of great interest in bone reconstruction, having demonstrated efficacy in both preclinical and clinical models [18,19,37,38]. These stem cells can be easily isolated and expanded in culture without ethical problems. Autologous transplantation can be performed with both cell types. AFSCs can be used in neonatal surgery to treat congenital malformation. DPSCs, available from puberty onwards, can be used to heal pathologies occurring in adult life.

The objective of this study was to investigate whether the application of collagen sponge scaffolds, combined with expanded and osteogenic precultured human stem cells, had an effect on vascularization and the osteogenic potential of the scaffolds *in vivo* after implantation into parietal bone defect of immunodeficient rats. We discuss which combination of scaffold/cells shows the highest potential for further investigations. Adequate porosity and surface properties are recognized as important parameters in identifying scaffolds for tissue engineering. Not fully interconnected and irregularly shaped pores often lead to insufficient vascularization [39]. Depending on the site of implantation, vascularization might be the critical step for successful tissue engineering.

The collagen sponge scaffold, currently used in several therapeutic practice, has been largely investigated in the literature – some authors have reported that it is not appropriate for successful new bone formation, because a mineral matrix, or a bone morphogenetic protein-expressing nonmineral matrix, or production of bone morphogenetic protein-2 by genetically engineered cells is also required [40]. However, our results contradict these previous data, because the cell-free collagen sponge showed an excellent healing bone process, especially at the longer time point. Our data therefore support the hypothesis that collagen membranes are a good scaffold, facilitating cell adhesion and bone-forming cells in the defect site.

However, our data show that cell-seeded scaffolds perform a better and faster bone reconstruction capability than collagen alone. This improvement can be due to both the osteogenic and the vascular differentiation potential of DPSCs and AFSCs, even if AFSCs seems to show a better result. Histological staining of human mitochondria confirmed the presence of human stem cells after 8 weeks of implantation in the new bone tissue and also in vessels, creating the endothelium of the new vessel network in the scaffold. A recent study showed potential for AFSCs in bone regeneration and angiogenesis in bone defects [41], while a demonstration of the role of AFSCs in both the processes was lacking. Our data demonstrate that human cells, present in the graft after 2 months, are also expressing osteocalcin inside the new bone and von Willebrand factor around vessels, being responsible for osteo and vascular formations. This may be due to the choice of implanting AFSCs after 1 week of osteogenic differentiation *in vitro*. In this condition osteogenic markers are not yet increased, and therefore a subpopulation of undifferentiated AFSCs could generate vessels, responding to local environmental signals. Vascularization of cell-seeded implants can play an important role for cell survival and differentiation in the scaffold, because these cells need oxygen to form osteoblasts.

The AFSC and DPSC populations employed here are c-kit^+^ and ckit^+^-CD34^+^, respectively, being superficial markers expressed in hematopoietic and vascular-associated tissue [42].

## Conclusion

Our findings support previous studies showing DPSC and AFSC angiogenic capability [30,43] and indicate the potential of these stem cells in several regenerative medicine strategies, such as bone reconstruction, providing vascularization of the implant.

## Abbreviations

AFSC: Amniotic fluid stem cell; BSA: Bovine serum albumin; DPSC: Dental pulp stem cell; H & E: Hematoxylin and eosin; MEM: Modified Eagle’s medium; PBS: Phosphate-buffered saline; PCR: Polymerase chain reaction; RT: Reverse transcriptase.

## Competing interest

The authors declare that they have no competing interests.

## Authors’ contributions

TM participated in the design of the experiment, carried out most of the experiment and drafted the manuscript. MR participated in the design of the experiment and carried out animal experiments. AP prepared *in vitro* cultures and carried out animal experiments. MZ carried out animal experiments and participated in drafting the manuscript. GC carried out animal experiments. FB carried out diaminobenzidine analysis. GBLS collected amniotic fluids and the informed consents of patients. AM helped to draft the manuscript and performed statistical analysis. ADP interpreted data and collected dental pulps and the informed consents of patients. All authors read and approved the final manuscript.

## Supplementary Material

Additional file 1 A table describing the three treatment groups of implants. Click here for file

Additional file 2 **A figure showing H & E staining of serial transversal sections (10 μm) of the whole cranial defect closed with collagen or collagen + AFSC constructs 8 weeks post surgery.** The 10 sections shown for each construct were obtained from the center (top) to the end (bottom) for embedded samples. The images demonstrate for both the shown samples that defects were completely regenerated with bone tissue after 8 weeks of implant. The same pattern was observed also for collagen + DPSC constructs (not shown). Click here for file

Additional file 3 **A figure showing human DNA detection in rat bone samples: both samples of collagen seeded with DPSC or AFSC react for human RNase P primers for DNA amplification in real-time PCR.** Right: reaction of a bone sample with a contamination of human cells, so a sample with a small amount of human cells. Method: Extraction Master pure complete (Epicenter); AB7000 platform (Applied Biosystem); Human RNase P primers and kit of amplification from RNAse P detection (Life Technologies, Paisley, UK). Click here for file

Additional file 4 **A figure showing DNA analysis with fluorescence hybridization *in situ*: a probe for human X chromosome (Abbott Molecular, Abbott Park, Illinois,USA) was used for checking the nuclei form human origin inside implants (red spots into the nuclei).** We can observe the staining in part of the cells present inside the new bone and vessels. Click here for file

Additional file 5 **A figure showing immunofluorescence for human nuclei: anti-human nuclei (anti-nuclei antibody, clone 235–1 MAB1281, species reactivity: human only; Millipore (Billerica, MA, USA) were used.** Anti-human nuclei (in red) co-localized with 4′,6-diamidino-2-phenylindole staining in some cells present inside the implant, demonstrating again the role of human cells in bone reconstruction. On the other hand, outside the implant area, no reaction occurred. Click here for file

Additional file 6 **A figure showing negative control for reactions with mouse anti-human mitochondria: image represents the reaction with anti-human mitochondria in a sample of I Group, without human cells seeded.** The image shows that the green signal (relative to h-mit) is very low, as a secondary antibody nonspecific signal. Click here for file
