# Supplementary material for: Human amniotic fluid-derived and dental pulp-derived stem cells seeded into collagen scaffold repair critical-size bone defects promoting vascularization
Source: Stem Cell Res Ther. 2013 May 21;4(3):53. doi: 10.1186/scrt203 (PMC3706961; doi:10.1186/scrt203)
Supplement: Additional file 2 — A figure showing H & E staining of serial transversal sections (10 μm) of the whole cranial defect closed with collagen or collagen + AFSC constructs 8 weeks post surgery. The 10 sections shown for each construct were obtained from the center (top) to the end (bottom) for embedded samples. The images demonstrate for both the shown samples that defects were completely regenerated with bone tissue after 8 weeks of implant. The same pattern was observed also for collagen + DPSC constructs (not shown). [file scrt203-S2.pdf]

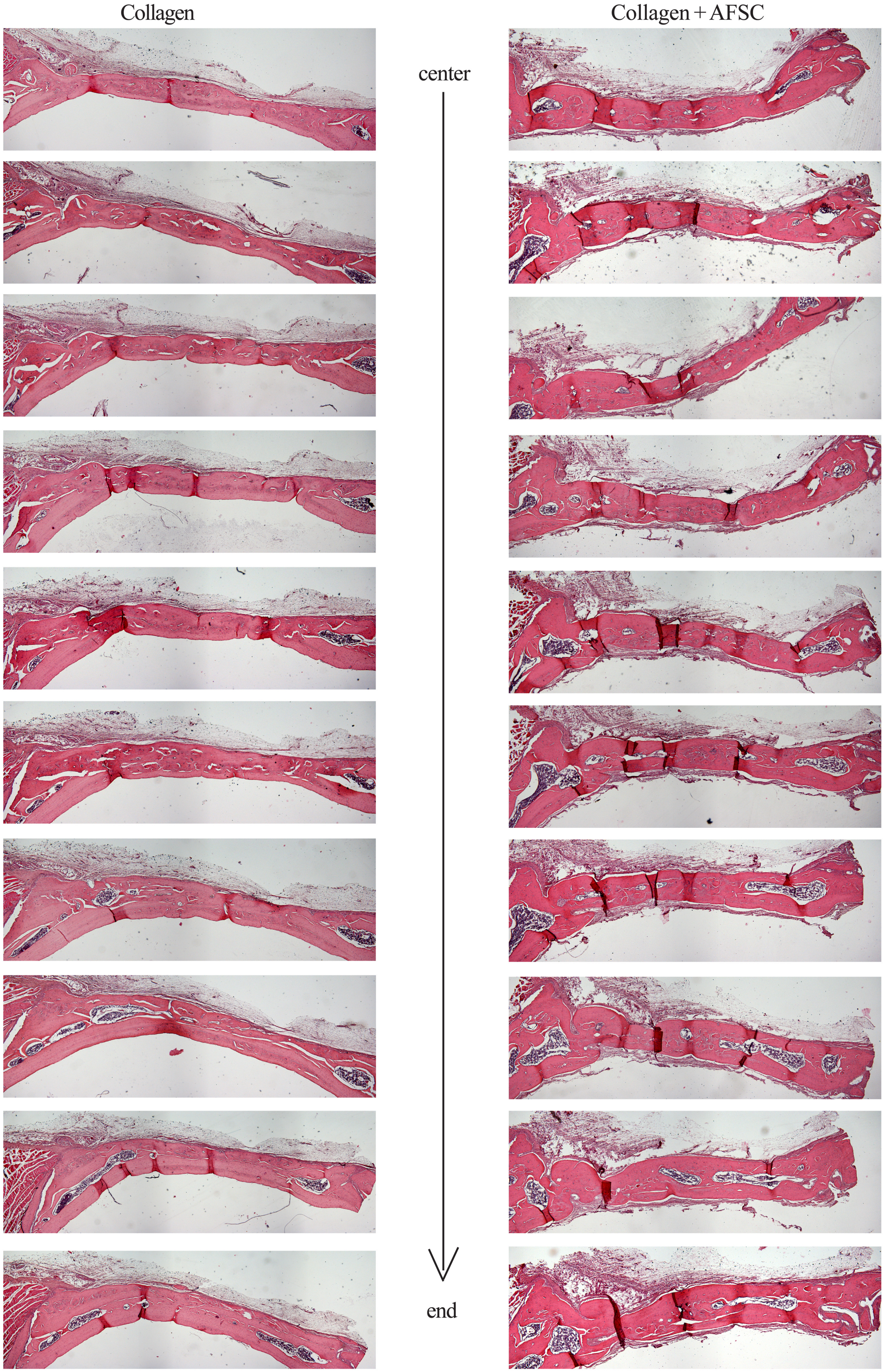

AD 1 Haematoxylin/eosin staining of transversal sections (10 μm) of the whole cranial defect closed with collagen or collagen + AFSC constructs 8 weeks post-surgery. The ten sections shown for each construct were obtained from the center (top) to the end (bottom) for embedded samples.
