# Supplementary material for: Human amniotic fluid-derived and dental pulp-derived stem cells seeded into collagen scaffold repair critical-size bone defects promoting vascularization
Source: Stem Cell Res Ther. 2013 May 21;4(3):53. doi: 10.1186/scrt203 (PMC3706961; doi:10.1186/scrt203)
Supplement: Additional file 3 — A figure showing human DNA detection in rat bone samples: both samples of collagen seeded with DPSC or AFSC react for human RNase P primers for DNA amplification in real-time PCR. Right: reaction of a bone sample with a contamination of human cells, so a sample with a small amount of human cells. Method: Extraction Master pure complete (Epicenter); AB7000 platform (Applied Biosystem); Human RNase P primers and kit of amplification from RNAse P detection (Life Technologies, Paisley, UK). [file scrt203-S3.pdf]

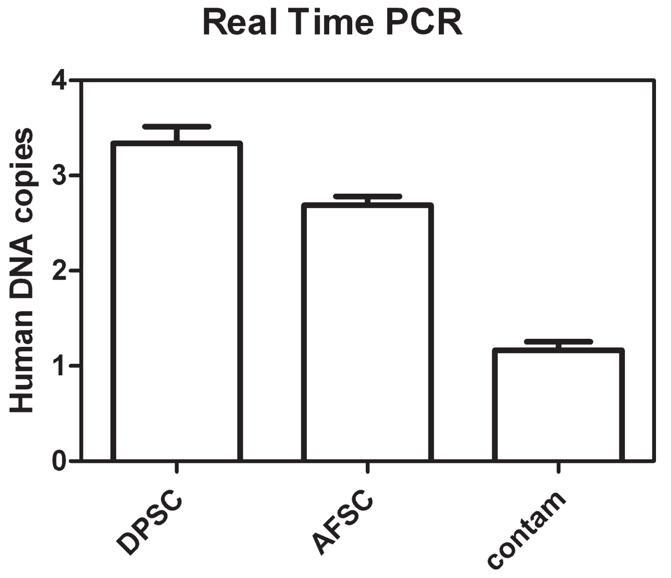

Figure AD2 - Human DNA has been found in rat bone samples: samples of collagen seeded with DPSC or AFSC did react for Human RNase P DNA amplification,. The arbitrary units expressed in the graph were obtained calculating the delta Ct with a positive (human) control. On the right a control of bone sample with a contamination of human cells, thus a sample with a small amount of human cells: the relative abundance of Human RNase P in this sample is lower than samples with DPSC or AFSC, indicating that the amplification observed is not only due to a possible contamination.
