# Supplementary material for: Human amniotic fluid-derived and dental pulp-derived stem cells seeded into collagen scaffold repair critical-size bone defects promoting vascularization
Source: Stem Cell Res Ther. 2013 May 21;4(3):53. doi: 10.1186/scrt203 (PMC3706961; doi:10.1186/scrt203)
Supplement: Additional file 4 — A figure showing DNA analysis with fluorescence hybridization in situ: a probe for human X chromosome (Abbott Molecular, Abbott Park, Illinois,USA) was used for checking the nuclei form human origin inside implants (red spots into the nuclei). We can observe the staining in part of the cells present inside the new bone and vessels. [file scrt203-S4.pdf]

DPSC implant **AFSC implant**

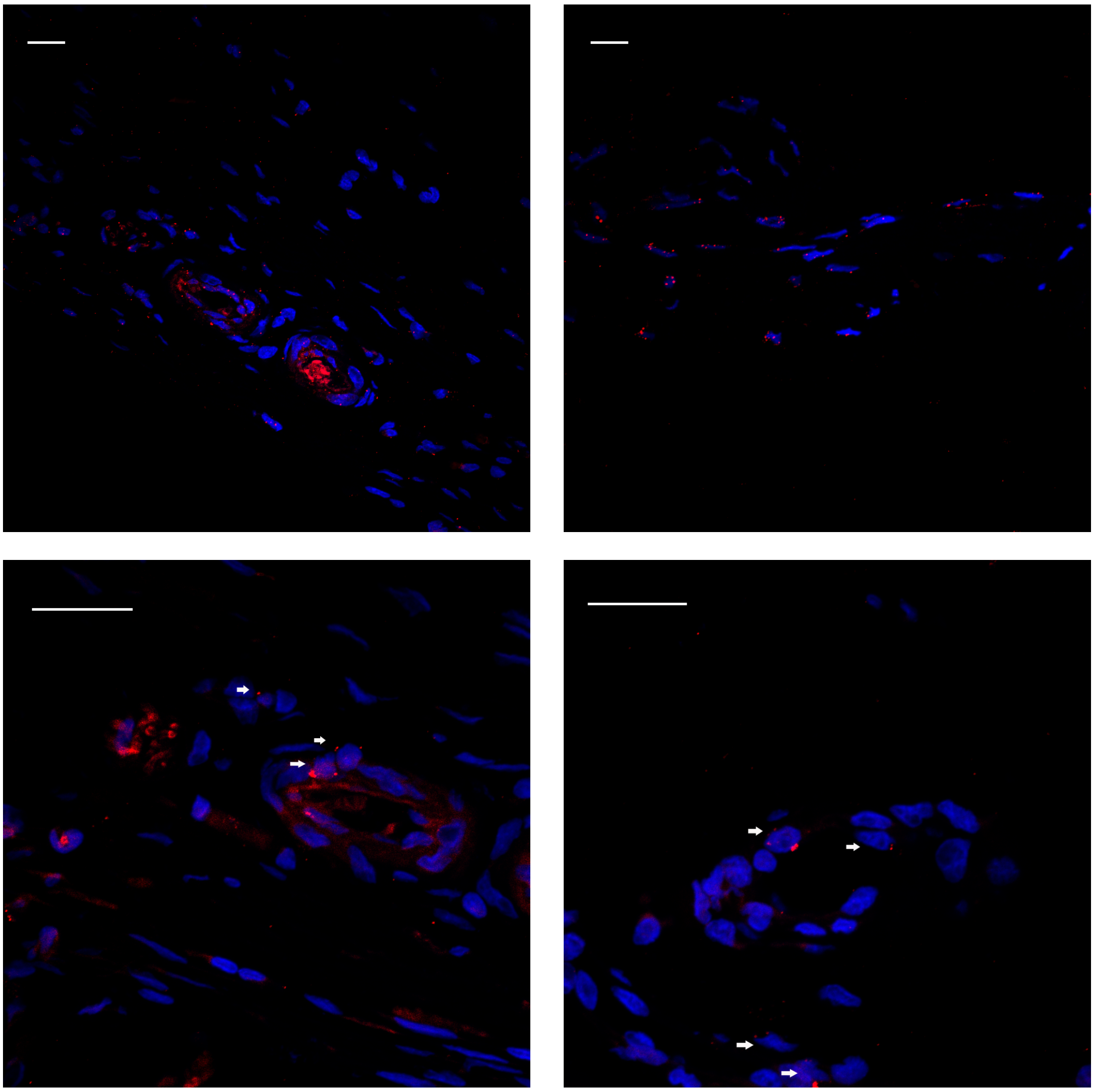

DAPI humanX chrormosome

AD3 - FISH analysis for X human chromosome - Confocal images of implants obtained 8 weeks after surgery. Double fluorescence signals from DAPI (blue) and anti-Human XC (red) Ab images. On the left new bone obtained after implant with collagen colonized with DPSC and, on the right, collagen colonized with AFSC. Images on the top were taken with a lower magnification. One o two pink spots inside the nuclei reveal the presence of human X chromosome (arrows). Scale bar= 30 µM
