# Supplementary material for: Human amniotic fluid-derived and dental pulp-derived stem cells seeded into collagen scaffold repair critical-size bone defects promoting vascularization
Source: Stem Cell Res Ther. 2013 May 21;4(3):53. doi: 10.1186/scrt203 (PMC3706961; doi:10.1186/scrt203)
Supplement: Additional file 5 — A figure showing immunofluorescence for human nuclei: anti-human nuclei (anti-nuclei antibody, clone 235–1 MAB1281, species reactivity: human only; Millipore (Billerica, MA, USA) were used. Anti-human nuclei (in red) co-localized with 4′,6-diamidino-2-phenylindole staining in some cells present inside the implant, demonstrating again the role of human cells in bone reconstruction. On the other hand, outside the implant area, no reaction occurred. [file scrt203-S5.pdf]

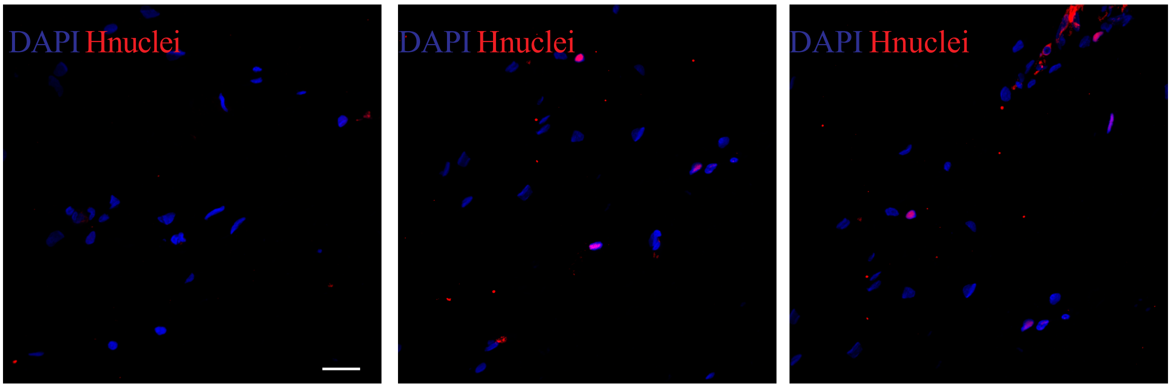

host bone DPSC implant AFSC implant

AD4 - Confocal images of implants obtained 8 weeks after surgery. Double fluorescence signals from DAPI (blue) and anti-Human nuclei (red) Ab images. On the left host bone: human nuclei labelling does not occur. In the middle: new bone obtained after implant with collagen colonized with DPSC and, on the right, collagen colonized with AFSC: pink signal reveals the labelling of anti-human nuclei. Scale bar= 30 μM
