# Supplementary material for: Human amniotic fluid-derived and dental pulp-derived stem cells seeded into collagen scaffold repair critical-size bone defects promoting vascularization
Source: Stem Cell Res Ther. 2013 May 21;4(3):53. doi: 10.1186/scrt203 (PMC3706961; doi:10.1186/scrt203)
Supplement: Additional file 6 — A figure showing negative control for reactions with mouse anti-human mitochondria: image represents the reaction with anti-human mitochondria in a sample of I Group, without human cells seeded. The image shows that the green signal (relative to h-mit) is very low, as a secondary antibody nonspecific signal. [file scrt203-S6.pdf]

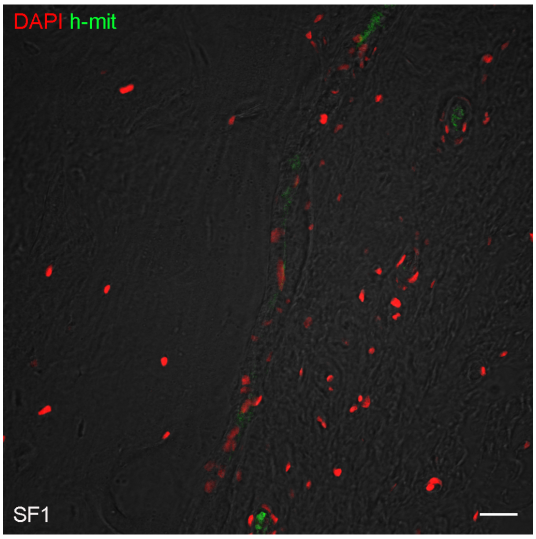

AD5- Confocal image of collagen implant alone obtained 8 weeks after surgery. Double fluorescence signals from DAPI (red) and anti-Human mitochondria protein (green) Ab image. Scale bar= 30 μM
